# Categorization for Faces and Tools—Two Classes of Objects Shaped by Different Experience—Differs in Processing Timing, Brain Areas Involved, and Repetition Effects

**DOI:** 10.3389/fnhum.2017.00650

**Published:** 2018-01-09

**Authors:** Vladimir Kozunov, Anastasia Nikolaeva, Tatiana A. Stroganova

**Affiliations:** MEG Centre, Moscow State University of Psychology and Education, Moscow, Russia

**Keywords:** visual perception, feature binding, magnetoencephalography, representational similarity analysis, visual processing stages, sensorimotor associations, value, repetition effects

## Abstract

The brain mechanisms that integrate the separate features of sensory input into a meaningful percept depend upon the prior experience of interaction with the object and differ between categories of objects. Recent studies using representational similarity analysis (RSA) have characterized either the spatial patterns of brain activity for different categories of objects or described how category structure in neuronal representations emerges in time, but never simultaneously. Here we applied a novel, region-based, multivariate pattern classification approach in combination with RSA to magnetoencephalography data to extract activity associated with qualitatively distinct processing stages of visual perception. We asked participants to name what they see whilst viewing bitonal visual stimuli of two categories predominantly shaped by either value-dependent or sensorimotor experience, namely faces and tools, and meaningless images. We aimed to disambiguate the spatiotemporal patterns of brain activity between the meaningful categories and determine which differences in their processing were attributable to either perceptual categorization *per se*, or later-stage mentalizing-related processes. We have extracted three stages of cortical activity corresponding to low-level processing, category-specific feature binding, and supra-categorical processing. All face-specific spatiotemporal patterns were associated with bilateral activation of ventral occipito-temporal areas during the feature binding stage at 140–170 ms. The tool-specific activity was found both within the categorization stage and in a later period not thought to be associated with binding processes. The tool-specific binding-related activity was detected within a 210–220 ms window and was located to the intraparietal sulcus of the left hemisphere. Brain activity common for both meaningful categories started at 250 ms and included widely distributed assemblies within parietal, temporal, and prefrontal regions. Furthermore, we hypothesized and tested whether activity within face and tool-specific binding-related patterns would demonstrate oppositely acting effects following procedural perceptual learning. We found that activity in the ventral, face-specific network increased following the stimuli repetition. In contrast, tool processing in the dorsal network adapted by reducing its activity over the repetition period. Altogether, we have demonstrated that activity associated with visual processing of faces and tools during the categorization stage differ in processing timing, brain areas involved, and in their dynamics underlying stimuli learning.

## Introduction

There is an emerging view that the brain mechanisms that integrate the different features of sensory input into a meaningful percept are shaped by the experience of the perceiver when learning to interact with objects of distinct categories (e.g., Reichert et al., 2014). Previous functional magnetic resonance imaging (fMRI) studies have given support to this view by demonstrating that the recognition of visual objects that differ in the way that they are typically interacted with, engage distinct cortical areas. Specific spatial patterns of neuronal responses were found for a number of categories of objects, including faces, animals, houses, places, tools, artificial objects, etc. (Ishai et al., 2000; Henderson et al., 2011; Collins and Olson, 2014; Bracci and Op de Beeck, 2016). For instance, it has been shown that there is an extended network of cortical regions of the ventral stream where faces induce significantly stronger activation than any other category of objects studied (Allison et al., 1999; Kanwisher, 2000). In contrast, a number of studies designed to test the relationship between the visual perception of objects such as tools and the sensorimotor experience for which they afford, has demonstrated that object associated regions involve areas in the dorsal visual stream and premotor system (Grèzes and Decety, 2002; Beauchamp and Martin, 2007; Schubotz et al., 2014).

It, however, remains unclear whether these differences in cortical topography are related to perceptual categorization *per se*, or instead reflect later processes of mental access to the meanings that follow object recognition. In order to distinguish between these alternative hypotheses we utilized the high temporal resolution afforded by magnetoencephalography (MEG), and adopted the three-stage processing framework (Del Cul et al., 2007). This framework suggests that “localized recurrent processing” for a time window 150–250 ms represents an intermediate stage between stimulus category insensitive low-level processing (<120 ms), and late stage of conscious awareness that is correlated with subjective report processing (after 270 ms). This intermediate stage is characterized by category-specific activity that contributes to the subsequent transition toward conscious access of representation, but does not yet correspond to a full-blown conscious state. In the present study, we pay special attention to this stage in order to investigate the neuronal processing that forms a consciously accessible representation through integration of the low-level features of sensory input. In particular, we aim to analyze differences in processing for two categories of objects which differ substantially in the types of experience normally associated with their interaction.

The ecological approach (Gibson, 1979) states that any act of perception is determined by both features of the agent and that of the environment. However, it remains unknown as to what the relative contribution of these features are and how they interact to give emergence to a perception. Some neurobiological models of perception such as Fagg and Arbib (1998) or Schubotz (2007) have suggested that object perception is based upon access to motor schemas pertaining to interaction with the object without explicit execution of the motor command. Behind such models there is the notion that perception has evolved as an associative appendage over a set of relatively independent visual control systems for the different motor outputs represented by independent pathways from the visual receptors through to the motor nuclei (Goodale and Milner, 1992). A binding mechanism based on sensorimotor associations is well suited for information integration when perceiving objects with a narrow set of predefined actions, such as tools, utensils, or sports equipment. In these cases, the environment informs executive mechanisms that constrain the way by which stimulus features may be integrated and represented by the organism for future use. However, for a large number of object categories, such as those involving living things, the possible set of interactions has a large degree of uncertainty and in these cases, direct association between sensory inputs and motor outputs seems to be an implausible mechanism of feature binding.

An alternative source of constraints originates from the neuronal dynamics of the organism itself. The linkage of neuronal groups by recursive exchange of signals across multiple, parallel, and reciprocal connections (Edelman, 1993), can lead to selective synchronization. This synchronized activity among neuronal groups can form coherent circuits corresponding to perceptual categories (Sporns et al., 1991). This mechanism is substantially different from a sensorimotor one. Firstly, it integrates the information by means of a system’s relaxation dynamics from perturbation caused by stimulus, to a state that represents a whole object. Secondly, features originating from self-organized dynamics, do not have a direct adaptive or functional meaning. They constitute how well the influence of a stimulus is suitable to sustain or suppress emergent dynamics of the system and so should be determined with respect to the system parameters.

Edelman and Tononi (2000) have suggested a model for a dynamical multi-dimensional neural reference space. Initial dominant axes of the reference space are related to dimensions that are concerned with the phenotypic aspects of organism itself, whilst incoming signals from the external environment (non-self) are assimilated with respect to these axes. Authors have related such constraining aspects, selected during evolution of organisms, to values and called the appropriate mechanism of perception as value-dependent one. The main difference between sensorimotor-based and value-dependent mechanisms of perception lay in the different roles that sensory information plays in their respective integrative processes. In the former, sensory input through sensorimotor associations guides execution of intentionally driven object recognition. For the functioning of this mechanism, it is important that one’s current task does not divert attention from the perception. In the latter, input from the external environment “feeds” the brain’s dynamics in a manner independent of the consciously performed task and “automatically” biases the competition between neuronal groups in favor of one representation over others. A winning state represents a whole object in the value-dependent reference space and simultaneously segregates it from the “background.” Sensorimotor features are determined in the terms of real-world manipulations and required to be independently representable (and later bound together in a part-to-part manner). A value-dependent mechanism instead integrates information across some incommensurable dimensions [e.g., valence, dominance (Koelstra et al., 2012), “time reference” (Bode et al., 2014)] and does not perform a linkage of separate representations.

These two mechanisms of categorization are manifesting different types of experience of the perceiver when learning to interact with objects. Sensorimotor experience is acquired by creating new, or reweighting of existing associations under the control of backward propagation of an error. Computation of the error between some target and the actual state does not require learning. Rather, it is the action itself, that allows for a transfer to the target state that must be learnt. Nevertheless, training is relatively straightforward and so learning occurs quickly because there is an explicit state that is the target of the action. In contrast, value-dependent experience does not have an explicitly representable target state. Instead, it changes parameters of dynamical system to set up a new stable state that depends both on persistent external influences and principles of self-organization (e.g., Bak et al., 1988). In other words, value-dependent experience is acquired through a prolonged training and does not result in getting good at reaching a desirable state but instead leads to a change in the value reference structure that makes an actual state more desirable. The long duration of an external influence is a principal but not exclusive characteristic that indicates the degree of value-dependent experience. Furthermore, the intensity of influences (as expressed by neural system arousal) determines how high in the hierarchy of values the experience can affect changes. The same effect is achieved when influences act earlier in the ontogenesis. Faces, such as those that we use as the stimuli in the study, are the percepts with the which the normal perceiver demonstrates a high level of expertise. They usually bring emotional content and skills in facial recognition are acquired soon after birth. We suggest that faces represent a category shaped by value-dependent experience. On the other hand, tools lack all of these characteristics and are instead learnt through functional manipulations making them a good example of an object category whose representation is dominated by the sensorimotor experience of interactions with them.

MEG affords recordings of a high temporal resolution that are well suited to isolating the spatiotemporal patterns of neuronal activity corresponding to different stages of visual object recognition. However, despite numerous efforts to improve accuracy of neuronal source localization using encephalography techniques (Wipf et al., 2010; Kozunov and Ossadtchi, 2015; Sohrabpour et al., 2016), the information gained in many experimental designs is insufficient to provide a comprehensive and robust estimate of the spatial distribution of the neural responses underlying perception of different classes of objects. A significant step toward solving this problem has been made through the use of multivariate pattern classification techniques for encephalographic signal analysis (Rieger et al., 2008; Ramkumar et al., 2013; Cichy et al., 2014; Wardle et al., 2016). The basic idea is to conceptualize experimental activity in multiple sensors as patterns and then discover statistical relationships between these patterns according to different experimental conditions. This turns the task into an immediate application of pattern classification techniques readily available from machine learning (Duda et al., 2000; Geurts, 2001).

The principal disadvantage of applications of pattern classification techniques is that raw classifier outputs are difficult to interpret. In order to work around this problem and extract components of neuronal activity specific for distinct brain functions, representational similarity analysis (RSA; Kriegeskorte et al., 2008) was introduced. The strength of RSA is that it allows to move from analyzing the accuracy of decodability of patterns themselves, toward an estimation of the relation of this decodability for the set of experimental conditions to some model dependencies expressed by representational dissimilarity matrices (RDMs). The application of RDMs provides a simple and universal language for asking questions about correspondence between relations among representations established by a hypothesized model and the relations among representations derived from the experimental data.

In the majority of cases where pattern classification methods for encephalographic signals have been applied, analyses were made in sensor space only. In some studies post-classification localization of regressor coefficients were used to obtain spatial patterns accompanying representation of particular brain functions in source space (Van de Nieuwenhuijzen et al., 2013; Clarke et al., 2015). Here we offer another way to extract sufficiently precise spatial maps from MEG signals. A fine-scaled distributed inverse solution is obtained before classification by use of the sLORETA inverse operator (Pascual-Marqui, 2002). Vertices are then combined into moderately coarse atlas-based regions and only three principal components of activities across each region are kept for training classifiers. This procedure preserves the majority of the variance available for each region and filters out the remaining noisy portion of it. In this manner, we obtain a method which allows for the estimation of a location and time-specific value of classification accuracy at every region on the whole cortex. Following this, we will then demonstrate that the subsequent application of RSA, which provides a contrast-based measure instead of absolute accuracy of decodability, and in combination with dimensional reduction of each regions to three principal components, give grounds to believe that obtained values are directly comparable between regions with a non-equal number of vertices.

In the present study using the MEG technique and applying the novel region-based multivariate pattern classification approach in combination with the RSA, we investigated spatiotemporal patterns of neuronal activity that underlie the formation of a meaningful percept for the two categories of visual stimuli. These categories, namely faces and tools, were chosen to be on the opposite ends of the spectrum according to the prevalence of either value-dependent or sensorimotor experience of interactions with them. While differences in processing of faces and tools have been repeatedly reported, it remained unclear whether these differences were related to categorization itself or to much later performing of object naming (Grèzes and Decety, 2002), dependent action (Beets et al., 2010), working memory operation (Linden et al., 2012), or introspection (Frässle et al., 2014). In this study, we made direct experimental testing of alternative hypotheses that associate the differences between brain areas engaged in processing of these categories either to intermediate processing stage of perceptual binding or otherwise to much later activation of response-related brain processes. In addition to this, we tested if procedural perceptual training has differential effects upon the mechanisms of feature binding associated with the perception of faces and tools. We interpret our results in a framework that assumes the existence of two different binding mechanisms for classes of objects differing by the prevalence of interactions that are determined upon either prior value-dependent experience or sensorimotor affordances.

## Materials and Methods

### Participants

Twenty two volunteers (10 males, 12 females) with an average age of 25.4 years (*SD* = 4.62) participated in the main experiment. This study was carried out in accordance with the recommendations of Declaration of Helsinki with written informed consent from all subjects. The protocol was approved by the ethics committee of Moscow State University of Psychology and Education.

### Stimuli

Overall during the main experiment, we used 26 bitonal (black and white) Mooney images. To produce them, we blurred ∼100 grayscale photographs of faces, animals, plants, and tools with a Gaussian filter and binarized them using a custom routine written in MATLAB (MathWorks, Inc.). Some nonsensical fragments of real grayscale photographs were also subjected to the same procedure. All resulting images were 500 × 500 pixels in size and equalized in luminance (number of white pixels) and length of the contours.

Following this, the images were shown to a group of 60 healthy volunteers, none of which would participate in the main experiment, in order to select a set of 26 images that matched the following characteristics: (A) two face, two tool, six animal, and six plant images should be correctly identified by more than 95% of subjects when seen for the first time; (B) two face and two tool images should be correctly identified by less than 15% of subjects when seen for the first time but should be correctly identified by more than 90% of subjects when seen after the corresponding original grayscale photograph had been shown; and (C) six nonsensical images should be identified as non-meaningful by more than 95% of participants.

The set of 10 images constituting the main subset of stimuli comprised: two simply recognizable faces, two simply recognizable tools, two naively unrecognizable faces, two naively unrecognizable tools, and two nonsense images. The remainder of the images constituted an auxiliary subset.

### Procedure

The stimuli were displaying to the participants using Presentation software (Neurobehavioral Systems Inc., United States) via a computer with a 60 Hz frame rate and were back-projected on a translucent white projection screen located 1.7 m in front of the participants to provide an 8 × 8 degrees visual angle.

The images were presented within four 16-min blocks separated by breaks of roughly 5 min each. All images from the main subset and four different images from the auxiliary subset were shown during each block. Every image from the main subset was presented 40 times during each block. Images from the auxiliary subset were displayed 15 times during each block. They were not intended to be analyzed in the study and were used to maintain subjects’ attention. Stimuli were presented in a pseudo-random order for duration of 800 ms with interstimulus intervals varied randomly from 1000 to 1500 ms.

After each stimulus presentation, participants were required to name aloud what they had seen in the picture. We did not give a hint of how to name any picture except in the case of the nonsensical images. We asked the participant to say “nonsense” or “nothing” when they were unable to recognize a meaningful object. All subject’s responses were written down by the experimenter and recorded on a dictaphone. Later, during an offline ascription of categories to participant’s responses, we treated all appropriate responses as correctly categorized (for example, “woman” in response to the presentation of a woman’s face was treated as a correctly recognized face category). Overall, the appropriate meaning was attributed to simply recognizable faces images in 99% of presentations, to simply recognizable tool images in 92%, and to nonsense images in 4%. Only trials with correctly categorized stimuli were analyzed. Participants were asked to delay their responses following a stimulus offset. We excluded trials where the subject responded earlier than stimulus had disappeared from the screen.

**FIGURE 1:**
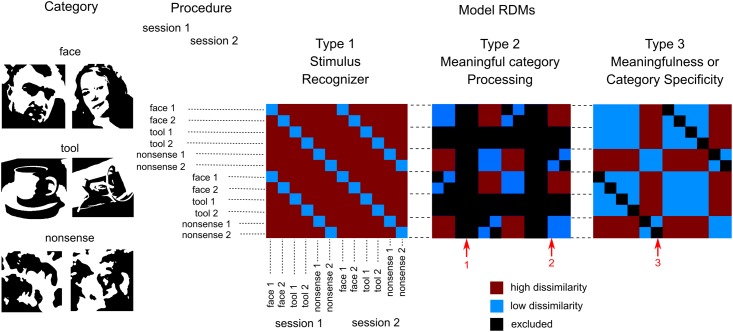
Design of the experiment and model RDMs used for analysis. We presented six images of three groups of stimuli (two in each group): two meaningful categories (faces and tools) and one that was nonsensical. Each image was presented by 40 times in a pseudorandom order in each of four blocks. Blocks 1 and 2 were combined to get 80 copies of each stimulus (first session), so as blocks 3 and 4 (second session). This provides 12 entries (6 images × 2 sessions) for the RSA analysis. Three types of model RDMs were used. Type 1: “Stimulus recognizer” that required all pairs of between stimuli dissimilarity to be high except the pairs between the same stimuli presented in first and second session. Type 2: “Meaningful category processing,” was used separately for face and tool categories. It required higher dissimilarity between patterns for stimuli across category boundary than within category boundary. Type 3: one group processing versus the other two, was used to extract either meaningfulness regardless of individual category differences or significant differences in one particular meaningful category processing. Red arrows indicate: 1—tool stimuli are excluded from “face versus nonsense” model (and vice versa); 2—low dissimilarity within category boundary; 3—pairs between the same stimuli in first and second sessions were excluded in type 2 and 3 RDMs. See text for detail.

Between second and third blocks we carried out a fast learning procedure during which a subject’s attention was guided to facilitate a recognition of the “naively unrecognizable” stimuli subtype. This procedure did not affect categorization of easily recognizable faces and tools, nor the nonsense stimuli. In the present study, we analyze only these three groups of images (total six images; **Figure 1**). Therefore, we suppose that the only effect influencing the processing of the stimuli reported here was perceptual learning gained through repeated viewing of the same stimulus.

### MEG Acquisition

Neuromagnetic activities were recorded with the helmet-shaped 306-channel detector array (“Vectorview,” Neuromag Elekta Oy, Helsinki, Finland). In this study, data from 204 planar gradiometers were used for analyses.

Prior to the MEG session, the positions of HPI coils were digitized together with fiducial points using the 3D digitizer “FASTRAK” (Polhemus, Colchester, VT, United States) and were used to assess a subject’s head position inside the MEG helmet every 4 ms. Later, offline position correction procedure was applied to the recorded data to compensate for a head movements. The mean change in the MEG sensor locations during the experiment ranged from 3 to 12 mm across subjects. The spatiotemporal signal space separation method (tSSS) implemented by “MaxFilter” (Elekta Neuromag Oy software) was used to suppress interference signals generated outside the brain. An electrooculogram (EOG) was recorded using four electrodes placed at the outer canthi of the eyes as well as above and below the left eye. The MEG signals were recorded with a band-pass filter of 0.1–330 Hz, digitized at 1000 Hz, and stored for offline analysis.

### MEG Data Preprocessing

The MEG data preprocessing was done using a combination of tools from SPM8 (Litvak et al., 2011), Fieldtrip (Oostenveld et al., 2011), and self-made routines in MATLAB environment. The data were converted into SPM8 format and epoched from 500 ms prior to stimulus onset and lasted until 1000 ms post-stimulus. Then information from the experimenters’ recorded observations were applied in order to select trials with correctly categorized images only and exclude trials with speech production artifacts. After that, data were low-pass filtered at 24 Hz, baseline corrected and merged to combine first and second blocks together as well as third and fourth blocks. The last step was done to provide two sections of 80 presentations (or less after artifact trial rejection) for each stimulus. Hereinafter these two sections are used to define two independent entries of every stimulus (see **Figure 1**).

Following this, an independent component analysis based artifact removal procedure was applied separately for each subject. A set of 10 random trials of each stimulus were drawn from both sections to produce a set of 60 trials and this set was decomposed into independent components. We visually inspected time courses, spectra and topologies of first 20 components and removed components comprising EOG, cardiac or muscle artifacts. After that, trials with residual artifact activity were rejected automatically using an algorithm that detects large deviations in the amplitude values based on adaptive thresholds for each person and channel.

### Source-Localization and Whole Cortex Atlas-Based Regions Definition

All results presented in this study are based on source-space analysis. To transfer the data into source-space, we applied an anatomically constrained inverse problem solver forcing the sources to lie on a tessellated mesh of the cortical mantle. The sources are considered as dipoles with fixed orientations normal to the local curvature of the mesh. The meshes were obtained on the basis of high-resolution structural T1-weighted MRIs acquired on a 1.5 T Toshiba ExcelArt Vantage scanner (TR = 12 ms, TE = 5 ms, flip angle = 20°, slice thickness = 1.0 mm, voxel size = 1.0 × 1.0 × 1.0 mm^3^). These structural scans were segmented and the gray-matter segment was used to construct a continuous triangular mesh representing the neocortex using FreeSurfer software (Dale et al., 1999).

The fiducial points digitized during MEG acquisition were then used to co-register the MEG and MRI spaces and meshes for every subject were downsampled to have 5002 vertices. These procedures as well as an overlapping spheres forward model and sLORETA inverse operator calculation were performed with Brainstorm software (Tadel et al., 2011).

In order to solve the problem of source co-registration across subjects, as well as to reduce a number of entries for the analysis, we used an automated labeling system for subdividing the human cerebral cortex into gyral and sulcus based regions as it is implemented in FreeSurfer. We chose Destrieux Atlas with 148 regions covering almost all cortical mantle (with an exception of some middle wall structures). Some small regions of this atlas were combined into larger ones (in the main in anterior regions of the cortex), while some oblong regions of temporal lobe were divided on posterior and anterior parts. Overall, we have obtained 82 atlas based regions. A list of these regions and their MNI coordinates of the seed vertices are given in **Table 1**. This table also contains the acronyms used from hereon to refer to the regions in the text. Every region of this list could consist of different number of vertices for each subject but represents the same anatomical structure. This allows for direct comparison of regional brain activity across subjects.

**Table 1:** List of all regions allocated in the study.

Region full name	Alias	MNI	Region full name	Alias	MNI
Frontal pole	FPole	25, 61, -5	Parahippocampal gyrus	PhG	18, -20, -22
Superior frontal	SF	18, 30, 44	Fusiform gyrus	FG	37, -58, -19
Middle frontal	MF	39, 40, 27	Medial occipito-temporal	MedOT	26, -53, -11
Inferior frontal	IF	50, 20, 11	Collateral sulcus anterior	ColSa	41, -25, -26
Orbital frontal	OF	23, 28, -16	Precuneus	Precun	6, -66, 47
Ventral premotor	VPM	40, 2, 34	Angular gyrus	AngG	49, -59, 40
Dorsal lateral premotor	DLPM	35, -10, 57	Inferior parietal gyrus	IP	57, -35, 31
Insula	Insula	40, 6, 1	Superior parietal gyrus	SP	21, -65, 59
Cingular anterior	CingA	3, 31, 23	Intraparietal sulcus	IPS	30, -60, 43
Cingular posterior	CingP	2, -26, 46	Parieto-occipital sulcus	POS	14, -69, 24
Postcentral gyrus	Postcen	43, -32, 49	Lunate sulcus	LunS	30, -90, 9
Central sulcus	Central	41, -16, 51	Cuneus	Cuneus	3, -83, 21
Temporal pole	TPole	32, 12, -38	Superior occipital sulcus	SOS	27, -84, 25
Superior temporal gyrus anterior	STGa	52, 8, -10	Superior occipital gyrus	SOG	16, -93, 34
Superior temporal gyrus posterior	STGp	55, -29, 16	Occipital sulcus anterior	OSa	44, -73, -1
Middle temporal anterior	MTa	65, -14, -20	Inferior occipital G and S	IO	36, -90, -10
Middle temporal posterior	MTp	67, -50, -2	Middle occipital gyrus	MO	39, -84, 19
Superior temporal sulcus anterior	STSa	52, -24, -6	Lingual gyrus	LingG	8, -67, 0
Superior temporal sulcus posterior	STSp	51, -61, 17	Calcarine sulcus	Calcarine	17, -70, 7
Inferior temporal anterior	ITa	58, -15, -28	Occipital pole	OPole	15, -101, -4
Inferior temporal posterior	ITp	50, -56, -20			


*The acronyms used in the text and MNI coordinates of the seed vertices are indicated. To denote a region in one hemisphere only, the prefixes “le” and “r” are used in the text for left and right respectively.*

### Pattern Classification

We used a MATLAB implementation of linear discriminant analysis (LDA) on the source-space transferred data. The time window used for analysis was taken from 100 ms prior to stimulus onset and lasted until 700 ms post-stimulus. Before classification, we resampled time courses with ratio 10:1 in order to reduce the number of entries for the analysis. This provided 81 time points with 10 ms resolution—the finest time resolution we report in this study.

To improve the signal to noise ratio, trials were averaged into pseudo trials. Every stimulus fell under the classification two times corresponding to the two sections, so the set of 80 trials (or less after artifact rejection) of any section was reduced to 10 pseudo trials by averaging a random selection of trials within this section. Each pseudo trial was an average of between five and eight trials. Conditions with less than 50 trials that were kept after artifact rejection were excluded from the analysis (overall seven such conditions were excluded across all subjects).

The input to the time-resolved classifier was specific for each brain region and consisted of the scores at a given time point for the three principal components of this region’s vertex time courses. Generalization of the classifier was evaluated using cross validation. For each pairwise comparison there were 18 trials used to train (nine from each stimulus class) and two used to test the classifier (one from each class). This procedure was repeated 100 times, each time with a new randomization. Classifier performance was quantified in terms of accuracy—proportion of correctly classified pseudo trials. The decoding analysis was run for all possible pairwise comparisons between stimulus patterns for each region and each time point.

We solved the inverse problem with 5002 vertices spatial resolution and only after that encapsulated data into the coarse scale regions. Moreover, we did not represent activity within a region by one component (averaging or taking one principal components) but kept three components for classification and only after that obtained a single measure for a region (and for each time point).

### RSA Model Definitions

We applied the RSA framework (Nili et al., 2014) to interpret results of pairwise pattern classifications. Within this framework, we constructed three types of simple model RDMs to separate information content specific for: (1) differences between individual stimuli; (2) differences between two groups of stimuli (of a meaningful category and nonsense stimuli) beyond those related to unique sensory features; (3) differences between one group and the combined other two. These RDMs were not intended to be comprehensive models of neural processing but represented model dependencies by values normalized to a range from 0 (low dissimilarity) to 1 (high dissimilarity). Relations between the empirical RDMs and the model RDMs were assessed by computing Spearman’s correlation coefficients between model and empirical RDM values separately for each region, each time point and each subject.

The first model, “Stimulus recognizer” (see **Figure 1**) required all pairs of between stimuli dissimilarity to be high excluding the pairs between the same stimuli presented in the first (blocks 1 and 2) and the second (blocks 3 and 4) sections. The “Meaningful category processing” model was applied separately for face and tool categories. Only subsets of pairwise comparisons corresponding to either “face versus nonsense” or “tools versus nonsense” pairs constituted RDMs in these cases (red arrow 1 on **Figure 1** indicates excluded pairs). This model type required higher dissimilarity between stimuli across category boundary than within category boundary (red arrow 2 on **Figure 1**). The third type of models were used to extract either differences between meaningful and meaningless stimuli regardless of their attribution to one specific category or instead differences specific to one particular meaningful category (face or tool) processing. It is worth noting that in type 2 and 3 models entries corresponding to the classification of the same stimuli across sections were excluded (red arrow 3 on **Figure 1**). Similarities between the same stimuli across sections are quite high, however, these similarities do not relate to category-specific structure. So excluding this information from the type 2 and 3 RDMs we improve SNR of the corresponding model relations. Whilst we kept entries corresponding to classification of the different stimuli across sections providing a generalization of the classification results.

### Effects of Stimulus Repetition

Sources of activity involved in the categorization are hypothesized to show amplitude modulation to repeated presentations of the stimuli. To investigate repetition effects for the activity of category-specific spatiotemporal patterns, we applied a similar procedure used for the classification analysis described above. All trials of a face stimulus for the first section were divided into 10 segments with five to eight trials in each of them (depending on the total number of trials available after artifact rejection) and then averaged within the segments. Unlike the classification analysis, in this case trials were taken in series, so that the first segment corresponded to the beginning of the section and the 10th corresponded to the end. We again took three principal components of evoked responses within the specific region but this time we superimposed them with the weights equal to the coefficients of the boundary equations from classification analysis. The motivation for this procedure was that the resulting equivalent current dipole’s time course represented activity within the region that best corresponded to category-specific activity. Following this, we averaged activity across both specific for categorization time interval and category’s exemplars. Absolute values of the obtained results were then subjected to a statistical analysis.

### Statistical Testing

Characteristics of raw classifier performance were tested using bootstrapping. We applied a bootstrap procedure that assigns labels randomly to trials in the first place. After finding the level where classification was better than chance, we tested the variability of the classification onset time as well as the peak times and accuracy values separately for different stimulus pairs and different brain areas. For each performance characteristic, we created 1000 bootstrapped samples by sampling participants with replacement. This yielded an empirical distribution. Setting *p* < 0.05, we rejected the null hypothesis if the 95% confidence interval did not include 0.

For statistical testing of the results represented by Spearman’s correlation coefficients we subjected all of them to Fisher transformation in order to perform variance stabilization. Following this, we used standard parametric statistical testing and ensured that distributions are close to normal. The model-specificity of spatial maps obtained by means of the proposed region-based RSA were estimated using multivariate analysis of variance (MANOVA).

In order to perform significance testing for the main part of the results we applied statistical parametric mapping (SPM, Friston et al., 1994). The main idea behind SPM is to consider an independent statistical model at each point in space and time and then use the general linear model framework to describe the variability in the data in terms of experimental effects and residual variability. Hypotheses expressed in terms of the model parameters were assessed at each region-time point with a univariate *t*-test. For the adjustment to the problem of multiple comparisons (82 regions × 81 time bins) we used an FDR corrected threshold (*p* < 0.01) for testing models of types 1 and 2 and a FDR corrected threshold (*p* < 0.05) for testing type 3 models. Any region-time point statistics that were below the threshold were treated as significant and we then determined the onset time (for a given region) as the first significant time point after stimulus onset. This approach was useful because we had predicted that some regions were activated in more than a single time window during the investigated interval. It would be difficult to discover such behavior with non-parametric cluster-based statistical approaches.

For the investigation of stimulus repetition effects, we used linear regression analysis for the amplitudes of signals corresponding to spatiotemporal clusters of category-specific activities. The hypotheses that a proposed regression models fit the data well were assessed using an *F*-test.

## Results

### Overview of Classification Performance

Decoding analysis was performed using LDA (Duda et al., 2000) for all possible pairwise comparisons between visual stimuli for each region-time point. Firstly, we examined whether decoding performance was above chance for classification performed on source-space data confined within different cortex regions. **Table 2** displays decodability characteristics averaged across all subjects for the five regions taken bilaterally in primary/secondary visual cortex, occipito-temporal lobe, parietal lobe, and premotor cortex. We have found that decoding accuracy was above chance beginning at 50–80 ms after stimulus onset for the majority of brain regions covering all cortex and for all category pairs. Following the initial onset of statistical significance, decoding performance rose to a peak and then decayed slowly but remaining above chance until stimulus offset for most regions.

**Table 2:** Subject level averages of the decodability characteristics for classification performed on source-space data confined within five brain regions (averaged across both hemispheres).

ROI	Classifier	Onset	Peak	Peak	Mean (*SD*)
		(ms)	(ms)	accuracy	
OPole	Face-nons	50	340	0.82	0.76 (0.053)
	Tool-nons	50	90	0.83	0.75 (0.049)
	Face-tool	50	90	0.85	0.76 (0.055)
	**All**	**50^2,3,4,5^**	**90^2,3,4,5^**	**0.83^∗∗^**	**0.76 (0.051)^∗∗^**
IO	Face-nons	60	150	0.82^∗^	0.72 (0.061)
	Tool-nons	60	320^∗^	0.76	0.70 (0.052)
	Face-tool	60	150	0.80	0.71 (0.053)
	**All**	**60^1,5^**	**150^1,5^**	**0.77^∗∗^**	**0.71 (0.053)^∗∗^**
FG	Face-nons	60	150	0.75^∗^	0.67 (0.044)
	Tool-nons	60	340^∗^	0.70	0.65 (0.041)
	Face-tool	60	150	0.73	0.66 (0.039)
	**All**	**60^1,5^**	**150^1,5^**	**0.70^∗∗^**	**0.66 (0.038)^∗∗^**
IPS	Face-nons	70	310	0.66	0.63 (0.034)
	Tool-nons	70	340	0.67	0.63 (0.034)
	Face-tool	60	330	0.66	0.63 (0.031)
	**All**	**70^1^**	**320^1,5^**	**0.66^∗∗^**	**0.63 (0.032)^∗∗^**
VPM	Face-nons	100	480	0.63	0.60 (0.026)
	Tool-nons	110	560	0.64	0.60 (0.038)
	Face-tool	70	360	0.63	0.59 (0.024)
	**All**	**80^1,2,3^**	**460^1,2,3,4^**	**0.62^∗∗^**	**0.60 (0.028)^∗∗^**


*Classifier characteristics for different stimuli category pairs as well as averaged across all stimulus pairs (in bold font) are shown. The onset time was determined as the first above chance decoding accuracy time point (FDR < 0.01). Mean accuracies were calculated on 50–700 ms time window. Numbers in superscript indicate the second region of the pair for significant differences in the order presented in the table. Single asterisks mark the characteristics that were significantly different between marked contrast and the other two within the same region. Double asterisks mark characteristics averaged across all stimulus pairs for a region and displaying significant differences for every between regions comparisons. See text for detail.*

Next we investigated whether decodability characteristics depend on a particular brain region. Classifier performance averaged across all stimulus pairs was examined. The significance onset time was determined as the first above chance decoding performance time point (FDR < 0.01). Mean accuracies were calculated for the 50–700 ms time window. Differences between regions at onset time, peak latency, and the maximum and mean accuracies were tested using bootstrapping. We have found that decodability onset time was significantly earlier for OPole than for all other regions and was later for VPM than for IO and FG. Peak latency replicated this pattern and was significantly earlier for OPole than for all other regions, whilst VPM was later than for all other regions. All pairwise differences of maximum and mean accuracies for tested regions were significant.

Finally, we examined the category specificity of the classification characteristics. In this case, and performed separately for each region, we used bootstrapping to test differences in decodability characteristics between different category pairs. None of the onset times nor mean accuracy differences were found to be significant. In IO and FG peak latencies for tool versus nonsense contrast were significantly later than for two other pairs. In IO and FG, the peak accuracy of classification for faces versus nonsense contrast was also significantly better than for the two other pairs. On the whole, we find that the performance of region-based classifiers is sufficient for reliable stimuli discrimination but is weak with relation to stimulus-class specific differences and largely uninterpretable.

### Can Region-Based RSA Provide Model-Specific Localization of Activity?

A given value of the accuracy of decodability between, for example, face and nonsense stimuli encapsulates differences in low-level visual processing, meaning-dependent binding and may be additional activity underlying response-related processing. In order to separate the constituents of an overall dissimilarity we applied the RSA technique (Nili et al., 2014).

**FIGURE 2:**
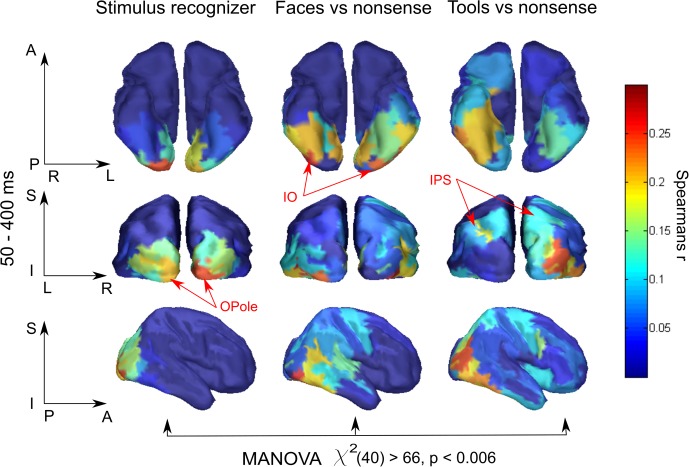
Brain region specificity of the model-related activity. Averaged across subjects spatial maps averaged for 50–400 ms are shown. Higher value of correlation in a region, expressed by Spearman’s correlation coefficient, means that within this region neuronal activity better fits to the function described by a corresponding model. Resulting maps show plausible localization: low-level processing prevails in occipital areas while meaning-specific processing is shifted in the anterior direction in category-specific way. Red arrows indicate characteristic regions for every model. MANOVA test result of model-specificity of spatial distribution is presented under the maps. See text for detail.

To our knowledge, this is the first application of RSA to MEG data computed in source-space and confined within separate regions in an attempt to obtain spatial maps of the model-specific activations of the cortex. Therefore, we examined whether the region-based RSA provided plausible localization of model activity. We took for every brain region, Fisher transformed Spearman’s correlation coefficients between MEG experimental data RDMs and three model RDMs: “Stimulus recognizer”; “face versus nonsense”; and “tool versus nonsense,” and averaged them across a time window at 50–400 ms (**Figure 2**). To make significance testing feasible we then averaged values across homologous regions of both hemispheres yielding 41 areas in total. We then performed a MANOVA to test whether the vectors of correlations corresponding to the 41 areas are significantly different for the distinct models. The analysis has revealed that means did not lie on the same line, i.e., the experimental data had different spatial structures of relations with all three models: χ^2^(40) = 66,63, *p* < 0.006. This provides evidence that distinct functional components extracted by applying different models have unique and persisting spatial distributions. Nevertheless, the results reported in this subsection are based on averages across long time intervals. In the following subsections, we move to describe results from a complete time-resolved analysis to investigate when and where a brain turns incoming sensory signals into a meaningful percept.

### Spatiotemporal Analysis Has Revealed Three Successive Stages of Visual Perception Processing

Here we report the results obtained by means of relation to “Stimulus recognizer” and “Meaningful category processing” models. The corresponding spatiotemporal clusters of neuronal activity have allowed us to outline three successive stages of visual perception processing. Some additional findings, confirming the category independent character of the third stage as well as the question of differences in the processing of the two meaningful categories we left until the next subsection.

**FIGURE 3:**
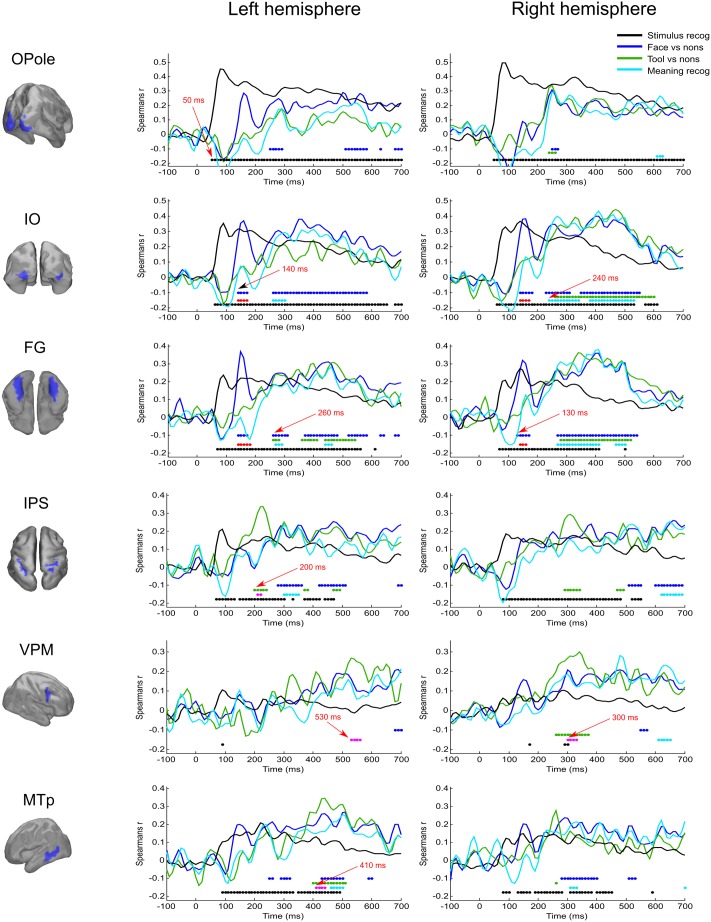
Averaged across subjects time courses of relation of activity in specified regions to four model RDMs. Each row represents results successively for left and right hemispheres for the localized region that is indicated in the left column. The common legend is applicable for all time courses: black line indicates time course of Spearman’s correlation coefficient with “Stimulus recognizer” model; blue line—“face versus nonsense” processing; green line—“tool versus nonsense” processing; cyan line—combined group of meaningful images versus nonsense stimuli processing. The discs of corresponding colors indicate time points where relations significantly (FDR < 0.01 for black, blue, and green, and FDR < 0.05 for cyan) differ from 0. Red discs indicate time points where face category processing is significantly different (FDR < 0.05) from both tools and nonsense stimuli processing; this face-specific activity was extracted through relation to the appropriate type 3 model (see **Figure 1** and text). Magenta discs indicate significant tool-specific activity (extracted in the similar way). Red arrows indicate some characteristic time points of significance onset.

Activity related to the “Stimulus recognizer” model in many aspects replicated the characteristics of raw classifier decodability that was averaged across all stimulus pairs. The significance onset time and peak time in posterior regions (see **Figure 3**, black lines) repeated the values for classifier accuracy and were 50–80 and 90–100, respectively. The spatial map of the extracted activity (see **Figure 2**) demonstrated a gradual decrease from the occipital pole regions in the anterior direction. This spatial distribution was very persistent in time and lasted without any changes from the onset time up to the end of the studied interval. In anterior temporal regions the onset time for “Stimulus recognizer” model was at ∼100 ms (60–80 ms for classifier for the same regions). Unlike for classifier accuracy, the correlation coefficients for “Stimulus recognizer” in the central and prefrontal regions did not exceed the significance threshold. Furthermore, the time courses of “Stimulus recognizer” outputs were more stereotyped: in the majority of regions, when the time courses exceeded a given threshold, they peaked very fast (not later 150 ms) and then slowly and monotonically decreased. This could speak in favor of the idea that the “Stimulus recognizer” extracts activity with simpler dynamics that we propose is attributable to a separate brain function which we presume to correspond to low-level sensory processing.

**FIGURE 4:**
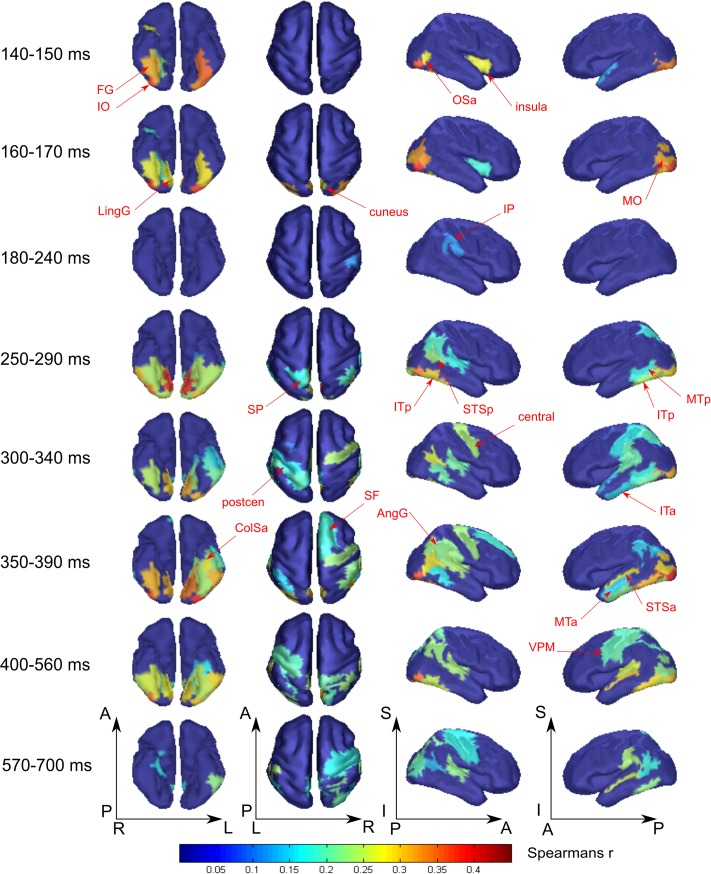
Averaged across subjects spatial maps of the relationship of measured activity to the “face versus nonsense” processing model. Successive time windows with persisted spatial distribution within each one are shown. Regions for which at least half of time points within the specified windows have significantly different from zero correlation coefficients are shown. The figure combines information of both significance and averaged value of correlation. Red arrows indicate the regions of the most prominent correlation to the model. Only newly appeared regions in the direction of increasing time are indicated.

The “Meaningful category processing” model characterizes the activity for which the categorical structure prevails over differences between individual stimuli. This has allowed us to determine the processing stage at which meaning-related visual feature binding is performed. We found that this stage began not earlier than 130 ms after stimulus onset (see **Figure 3**, blue lines). At this point activity of rFG was significantly correlated with the “face versus nonsense” processing model (**Figure 4**). Following this, in the time interval at 140–170 ms, a set of occipito-temporal regions of ventral visual stream were active, with the maximum in bilateral FG, IO, and OSa. Right insula also demonstrated the model-related activation for this interval. Cunei and MO in both hemispheres joined the previously indicated regions ∼20 ms later. From 190 to 240 ms, face category processing did not reveal itself in any place on the cortex (except rIP where some weak model-related activity in the interval 220–240 ms was found).

At 250 ms, an extensive network of the same occipito-temporal regions as found within the 140–170 ms window were supplemented by bilateral ITp, leMTp, leSP, rSTSp, and rAngG which started to distinguish faces from nonsense stimuli processing. A 50 ms following this, model specific activity was seen in left anterior temporal, motor and premotor areas which was then followed by a number of frontal regions, leading to the development of the distributed network that is thought to underlie meaning related response formation. By 600 ms after stimulus onset (but 200 ms before its offset) activity in ventral visual streams ceased to discriminate faces from nonsense stimuli processing. Only some lateral temporal, parietal and motor regions continued to discriminate between classes of stimuli.

**FIGURE 5:**
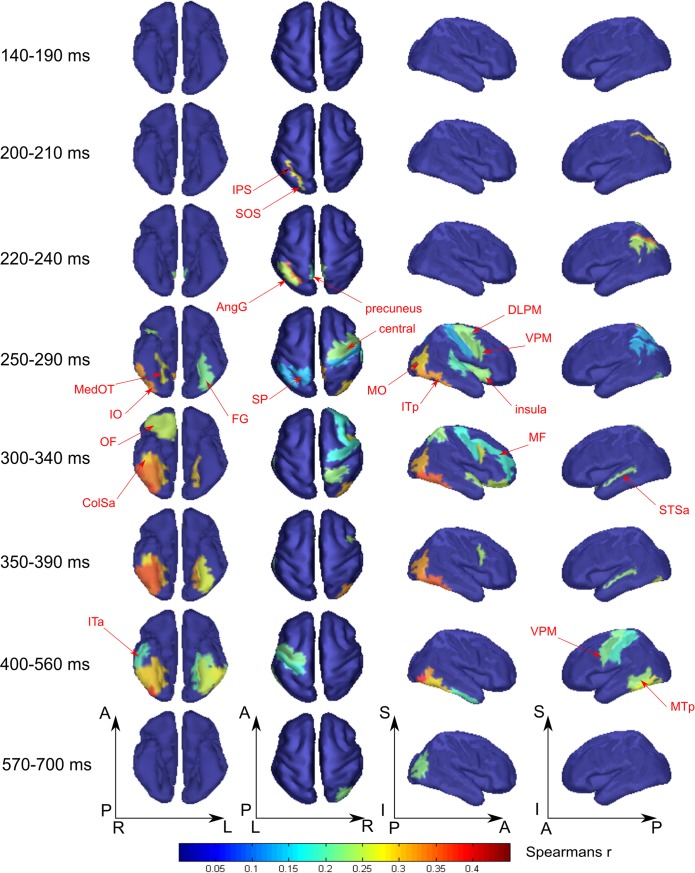
Averaged across subjects spatial maps of relationship of measured activity to the “tool versus nonsense” processing model. We kept for the most part the same grouping of time-points in the windows as for **Figure 4** except for combining 140–190 ms in one window while showing separately 200–210 and 220–240 ms intervals. Regions for which at least half of time points within the specified windows have significantly different from zero correlation coefficients are represented only. The figure combines information for both significance and averaged value of correlation. Red arrows indicate the regions of the most prominent correlation to the model. Only newly appeared regions in the direction of increasing time are indicated.

**Figure 5** displays the spatial maps of regions with significant differences between the tool and nonsense stimuli processing. The most prominent effect seen here is that the significance onset time of tool category processing was never earlier than 200 ms after stimulus onset (see also **Figure 3**, green lines). At this moment two areas of the left dorsal visual stream, leSOS and leIPS, started to significantly correlate with the “tool versus nonsense” processing model. After another 20 ms, leAngG and precunei in both hemispheres joined leIPS while leSOS ceased to be related to the model.

As was similar in the case with face processing, at 250 ms a large set of brain structures of ventral visual pathway (namely ITp, FG, and especially MedOT) started to differentiate between tool and nonsense stimuli processing. Additionally, the parietal, motor, and premotor areas showed some model-specific activity at this time. After 300 ms, some frontal regions of right hemisphere also began to demonstrate meaning-specific activity. It is worth noting that tool category processing appeared first in the left hemisphere but in the time interval at 250–400 ms processing shifted to the right hemisphere. However, after 400 ms it largely returned to the left hemisphere. By 600 ms, no activity in the cortex was able to discriminate tool from nonsense stimuli processing. This effect was even more pronounced in processing of tool stimuli than that of faces.

### What Is Specific for Particular Meaningful Category Processing and What Is Common for Meaning Formation?

**FIGURE 6:**
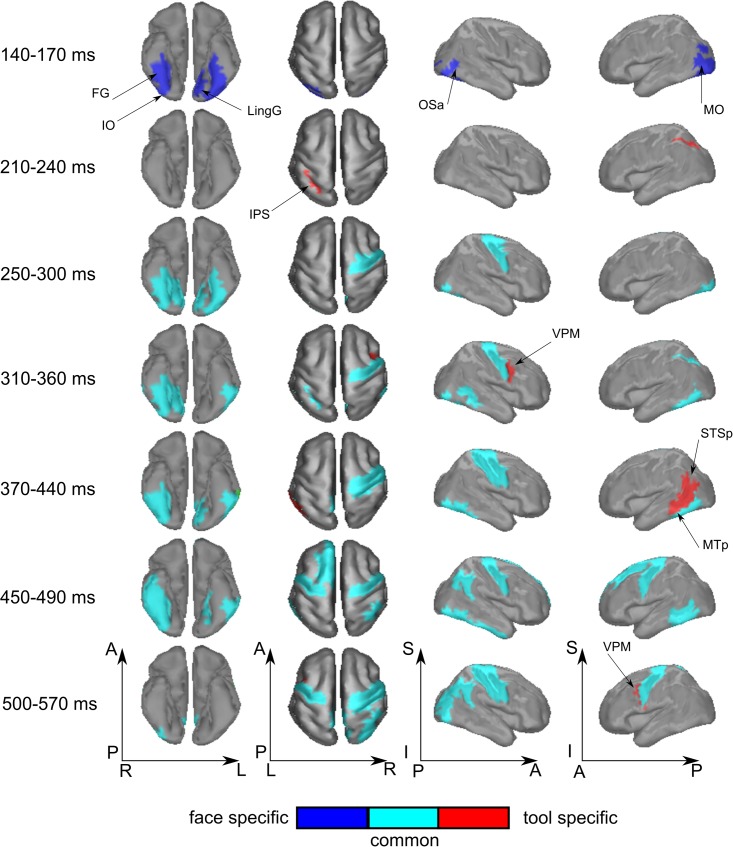
Averaged across subjects spatial maps of regions with specific for face or tool processing, as well as common regions for meaningful stimuli processing regardless of their attribution to a certain category. Successive time windows with persistent spatial distribution within each one are shown. Regions for which at least half of time points within the specified windows have significantly different from zero correlation coefficients are represented only. The figure (unlike **Figures 4**, **5**) does not provide value of correlation information. Instead, by color, we code either common or specific activity—please refer to the legend. Arrows indicate regions with significant meaning-specific differences between faces and tools processing.

The type 2 models do not allow the definition of activity that is specific for the processing of a certain category of meaningful stimuli. Therefore, to examine significant distinctions between face and tool category formation we applied the type 3 (see **Figure 1**) models. These models are similar to type 2 but represent all stimuli together, and treat the stimuli of an alternative meaningful category as the stimuli of “nonsense” group. Application of type 3 models has allowed us to avoid the detection of total differences between the processing of two categories (as if we used RDM to recognize directly between face and tool categories) and to analyze only that which are relevant for meaning-specific activity. **Table 3** lists all brain regions and time intervals for which a category-specific activity was detected (see also **Figure 6**).

**Table 3:** A list of all regions and time intervals with significant distinctions between processing of two meaningful categories.

Region	Time window (ms)
**Face-specific**	
leIO	140–170
rIO	140–170
leFG	140–180
rFG	140–160
leLingG	140–170
leMO	140–170
rLunS	150–170
leOSa	150–170
rOSa	160–170
**Tool-specific**	
leIPS	210–220
rVPM	300–330
leMTp	410–440
leSTSp	410–440
leVPM	530–560


*These spatiotemporal patterns were extracted through relation to the appropriate type 3 model (see **Figure 1**) which require one meaningful category processing is significantly different (FDR < 0.05) from both another meaningful category and nonsense stimuli processing.*

We have found that all face-specific activity happened within interval of 140–170 ms after stimulus onset. It was most pronounced throughout this period in IO (also known as occipital face area), and FG comprising fusiform face areas. Also, face-specific processing exceeded the significance threshold in leLingG and leMO at the same interval and in bilateral OSa and rLunS 10–20 ms later.

We have detected tool-specific activity before 250 ms and also at later intervals. The leIPS for 210–220 ms window demonstrated both high statistical significance and a high value of correlation with the corresponding RDM. Following 250 ms after stimulus onset, rVPM was indicated in the 300–330 ms window; as well as also the leMTp and leSTSp for the 410–440 ms window where we observed a moderate value of correlation to the model that narrowly exceeded threshold. In contrast, leVPM for the 530–560 ms interval had a moderate statistical significance but low value of correlation. It is worth noting that altogether these regions match exactly with the well-known network of tool preferring regions of Beauchamp and Martin (2007).

In order to examine the opposite problem of investigating the spatiotemporal structure common to processing of both meaningful categories of objects, we again tested the relation between measured data and the type 3 model, but this time we forced the dissimilarity to be low within the group by combining both the meaningful categories. In **Figure 6**, the cyan colored patches indicate regions common to processing of both meaningful categories and for successive time windows with persistent dynamics within each one (see also **Figure 3**, cyan lines).

At 250 ms after stimulus onset, activity common to meaning formation started to be significant. Some adjoined regions in occipito-temporal areas of right hemispheres exceeded the significance threshold and remained active for another 200–250 ms. For the homologous regions of the left hemisphere, the significance onset time (where it was applicable) was delayed for 20–30 ms with respect to the onset time in right hemisphere. At 280 ms, motor–premotor areas of the right hemisphere started to be significant and this state was maintained until the end of the entire interval that was analyzed. The activity of motor–premotor areas of the left hemisphere exceeded the threshold only ∼200 ms later than the right. Overall, in this time period the number of significant regions in right hemisphere was about two times greater than in the left one. Comparing **Figures 4**–**6**, it is easy to see that most of the significant spatiotemporal patterns after 250 ms were common for all three models. This suggests that these networks formed after 250 ms following stimulus onset are an essential part of meaning-specific processing that is performed in a category independent manner. We interpret this finding as a manifestation of the beginning of the third stage of visual perception processing starting around 250 ms. From hereon we refer to this third stage of perception as “supra-categorical” processing.

### Different Effects of Stimulus Repetition in the Category-Specific Spatiotemporal Patterns of Activity for Faces and Tools

We tested here for the existence of repetition effects for the activity of category-specific spatiotemporal patterns.

Based on the above results (for analyses that were independent of amplitude changes in response to the repeated presentation of the same stimulus) we have chosen activities in IO and FG regions of both hemispheres within the 140–170 ms window as the face-specific patterns related to binding processes. We have found that amplitude of activity of the chosen spatiotemporal patterns in the left hemisphere did not demonstrate significant repetition effects: leIO, regression coefficient *k* = 0.05 [*F*(1,218) < 0.08, *p* > 0.75]; leFG, *k* = -0.01 [*F*(1,218) = 0.007, *p* > 0.9]. However, in the right hemisphere both regions demonstrated significant repetition amplitude enhancement: rIO, *k* = 0.32 [*F*(1,218) > 4.5, *p* < 0.04], rFG, *k* = 0.21 [*F*(1,218) = 5.6, *p* < 0.02].

A similar analysis was conducted for the activity in the time window 210–220 ms for the IPS region of both hemispheres (tool-specific activity was found only in the left hemisphere but we made symmetrical analyses for the sake of completeness). We have found that unlike repetition enhancement in the regions of ventral stream for face evoked responses, there was a significant repetition suppression effect for processing of tool stimuli in the dorsal region of the left hemisphere (leIPS): *k* = -0.11 [*F*(1,218) > 4.3, *p* < 0.04]. No significant repetition effect was found in the right hemisphere: *k* = -0.02 [*F*(1,218) < 0.15, *p* > 0.7].

## Discussion

The present study aimed to characterize how category structure emerges in spatiotemporal patterns of brain activity and to determine the role of distinct neuronal mechanisms responsible for feature binding, dependent upon different object categories. To address these issues, we applied RSA and tested simple models by evaluating their ability to explain region-based time-varying neural activity patterns in MEG data. We used two meaningful image categories, namely faces and tools, that were chosen to be on the opposite ends of a spectrum relating to the prevalence of either value-dependent or sensorimotor experience based interactions with objects of each class.

From our results, we draw several major conclusions. The first outcome is methodological in nature. We verified that our novel method of region-based pattern classification analysis of MEG data allows for exploration of the spatial distribution of brain activity without the need for a priori spatial constraints. Our results are comparable to the localization of brain activity in previous studies using fMRI and similar experimental conditions. This consistency of sparse localization patterns provides a cross-validation for the spatial resolution of the method. Additionally, the vastly improved time resolution afforded by MEG signals makes it uniquely suited for investigation of highly temporally dynamic brain processes.

Secondly, the overall structure of the emergence of a meaningful percept matched very well with the notion of three stage processing (Del Cul et al., 2007). The earliest stage of cortical processing, represents low-level visual data analysis that distinguishes between any stimuli and is started at 50 ms following stimulus onset and has maximal decodability accuracy at 90 ms, the latency of the P1 component. Category-specific processing, as revealed by relating the data to an appropriate model requiring both within category similarity and between category dissimilarity, started no earlier than 130 ms after stimulus onset. The final stage observed in the study, reflected meaning processing regardless of attribution of the object to a specific category and started about 250 ms after stimulus onset.

Thirdly, we observed two heavily distinct spatiotemporal patterns of brain activity underlying the second stage of visual processing, that concerned the perceptual categorization of faces and tools. The face specific pattern discriminating the faces from both the tools and nonsense stimuli was related to bilateral activation of ventral occipito-temporal areas at 140–170 ms, while the tool-specific activation was shifted to the later time window of 210–220 ms and comprised IPS of the left hemisphere. Below we will speculate about potential nature of face specific binding processes and suggest that tools, because of insufficiency of such processing for this category, require later in time an additional procedure that completes groupings between separate segments to form a coherent representation of an object.

The final conclusion is based on the remarkable finding that activity within face and tool specific spatiotemporal patterns have different dynamics following procedural perceptual learning. While tool-specific activity demonstrates common decrease in amplitude as the perceptual task becomes easier, face-specific activity shows an opposite effect in that amplitudes increase as the stimuli become learned. If we assume there is a relation between face-specific processing and value-dependent mechanism of categorization, and tool-specific with a sensorimotor mechanism, then this result would indicate that these two mechanisms differ not only by localization in the brain and timing but also by differential dynamics in their learning processes.

### Region-Based Pattern Classification Analysis of MEG Data Provides Both Plausible Spatial Localization and Millisecond Resolution for Spatiotemporal Exploration of Neural Processes

Recent studies using RSA have characterized the spatial patterns of brain activity in the processing of distinct object categories through their relationship to computational models (Kriegeskorte et al., 2008; Raizada and Connolly, 2012; Anderson et al., 2016). Nevertheless, the fine temporal dynamics of neural processes that the brain uses to form meaningful representations of objects remains largely ignored in this area of research because of the predominant use of fMRI data as an input for RSA. It is believed that current brain imaging techniques in isolation cannot resolve the brain’s spatiotemporal dynamics because they provide either high spatial or temporal resolution but not both. fMRI-MEG data fusion has been proposed as a possible solution of this problem (Cichy et al., 2016).

Here we offered another method that provides a way to extract sufficiently precise spatial maps exclusively from MEG signals. Firstly, a fine-scaled distributed inverse solution was obtained before classification. Following this, vertices were combined in moderately coarse atlas-based regions and only three principal components of activities across each region were kept for training classifiers. The information of these components for every region preserved spatial specificity but was less sensitive to inaccuracies arising from the inverse solution. These were then submitted for time-resolved classification. Our method is most similar to the spatiotemporal searchlight approach (Su et al., 2012) but we suggest that it is more practical to implement. Firstly, it solves the issue of across subject co-registrations of analyzed spatial entries in a straightforward way. Secondly, it makes an application of statistical parametric tests more feasible because it notably reduces the number of spatial entries and makes multiple comparison correction less demanding. This property is useful because, as we have shown, some regions were activated during more than a single time window and thus it would be difficult to discover such behavior using approaches based upon non-parametric cluster-based statistics.

The results presented here provide clear evidence that novel pattern classification approach affords the capacity to go beyond spatially limited region-of-interest analysis using MEG data, and allows for spatially unbiased exploration of neural processes. The “Stimulus recognizer” and “Meaningful category processing” models have shown plausible spatial distributions of activity that are unique to each model. Neural activity first processed in the primary cortex is unique for all stimuli, with later activity then spreading in the anterior, either ventral or dorsal directions for faces and tools respectively. Regions displaying correlation with models extracting activity specific for a certain meaningful category processing have revealed a striking overlap with regions discovered using fMRI. The most distinguished face specific regions, displaying both significant and high-valued correlation coefficients, were represented by bilateral IO, as known as occipital face area, and FG comprising fusiform face area (Haxby et al., 2000; Palermo and Rhodes, 2007; Rossion et al., 2011). Significant tool specific regions were localized in the left IPS, MTp, and VPM, a well-known network displaying tool preferred activations (Chao and Martin, 2000; Lewis, 2006; Beauchamp and Martin, 2007). This cross-validation for the spatial specificity of the method strongly widens the applicability of MEG based studies to the pursuit of scientific questions without the need for spatial priors.

### Three Successive Stages of the Processing Underlying Visual Perception

In order to distinguish which one of the two alternative processes, perceptual categorization or later response-related brain procedures, causes the previously reported differential spatial patterns of neuronal responses underlying faces and tools perception it is necessary to separate the spatiotemporal clusters of neuronal activity corresponding to different stages of visual object identification. In this study, applying temporally and spatially unbiased exploration method, we observed three spatiotemporal clusters of neurofunctional activity corresponding to low-level features analysis, category specific feature binding and category independent meaning processing. This three stages scheme matches well to the one that was first proposed for face processing (Bruce and Young, 1986; Latinus and Taylor, 2006), and has subsequently been expanded for any category of objects (Del Cul et al., 2007).

Currently available information regarding the onset of cortical processing indicates that the earliest moment at which signals from retina passing the lateral geniculate body reach the cortex is about 40–50 ms after stimulus onset. This matches the latency at which MEG data start to distinguish between individual stimulus patterns in sensor space (Cichy et al., 2014; Wardle et al., 2016). Here we have replicated this result but based on source-space data confined to particular regions within the cortex. The activity related to the “Stimulus recognizer” model started to manifest itself at 50 ms after stimulus onset in primary/secondary visual regions. It reached a peak at 90 ms and then monotonically decreased. In the spatial sense, it gradually decreased toward the anterior direction preserving its spatial distribution during the whole period of processing and largely restricted to the occipital lobe. This activity profile speaks in favor of the predominantly low-level feature processing extracted through relation to the “Stimulus recognizer” model. Direct evidence that brain activity at the time of early cortical evoked responses represents low-level visual features processing has been introduced in a recent MEG study by Ramkumar et al. (2013) in which processing of spatial frequencies and orientations were separately extracted and exact onset time of activity corresponding to these features were discerned: 51 ms for spatial frequencies and 65 ms for orientations.

The grouping capabilities of the first stage are only necessary prerequisites for perceptual categorization because by themselves they cannot establish a link between the statistics of sensory input (Dumoulin and Hess, 2006) and the meaning that defines an object’s placement within a category. Ultimately, the fundamental question of how an emergent property such as meaning could arise from sensory data should be considered within meta-system frameworks like phase transitions and critical phenomena models (Kozma et al., 2014) or gauge theory (Sengupta et al., 2016). Tracking the exact details of the process by which spatial statistics of sensory input are transformed into a space invariant and meaningful percept was beyond the scope of the experiment. We aimed to investigate characteristics on the macroscopic scale and investigate the cortical localization as well as time deployment of neuronal activity corresponding to emergence of meaningful categorization. To this end, we applied a “Meaningful category processing” model that captured particular category membership by controlling higher dissimilarity between patterns for exemplars across category boundaries rather than within the category boundary. We found that the second stage of visual cortical processing, which is responsible for the meaningful binding of spatial image statistics, lasted for a time period approximately from 130 to 240 ms after stimulus onset and involved some regions of occipito-temporal and parietal areas without engaging primary visual cortex.

It is worth noting that the application of an appropriate model within RSA approach is a necessary but not sufficient condition for correct extraction of activity corresponding to meaningful category emergence. There are some experimental studies using the similar RDM (Carlson et al., 2013; Cichy et al., 2014) that reported much earlier onset times of distinguishability between categories. We believe that the reason for this group’s finding of such early onsets of category-specific processing is that in these cases there was limited control for differences in low level processing within categories. For example, in Cichy et al. (2014), 12 natural images of faces and bodies, that were similar in shape and color, were used to control similarity within respective categories. We argue that this is not sufficient to eliminate the effect that similarities in the image statistics may give rise to spurious group separation independent of its meaning. As the resulting corresponding model performed mostly as a stimulus differentiator and showed that distinction between face and body categories was possible from 56 ms, the latency at which individual images started to be distinguishable. In the present study, we used two bitonal images of each category sharing few common low-level image statistics (even Mooney faces are badly recognized by computer algorithms based on sensory data). Note negative deflection of “Meaningful category” model time traces (**Figure 3**) in the interval approximately from 50 to 120 ms post-stimulus, where dissimilarity between all stimuli prevails over categorical structure. This deflection confirms that in our case category-specific activity was carefully controlled to avoid influences from low-level processing. After careful extraction of activity corresponding to perceptual categorization stage, we found this state did not start earlier than 130 ms after stimulus onset.

Complementary evidence in favor of this hypothesis was presented in a recent study of Clarke et al. (2015) who demonstrated that meaning begins to be represented in visual processing during the second stage by showing that classification of individual images is significantly improved by adding semantic information from 200 ms after stimulus onset. The authors’ interpretation suggests that a model of object representations based on combination of sensory (derived from HMax model) and semantic measures provides a better account of underlying brain activity at this point than visual statistics alone. This result is in accordance with the line of evidences we present here: that meaning should start to be essential for explaining the brain activity during the perceptual categorization stage.

One important precaution should be taken when interpreting our results. We are not providing a complete list of regions that may contribute to processing of object categorization but only those structures that operate in a way independent of retinotopy. This is an inevitable consequence of the high selectivity of the “Meaningful category processing” RDM analysis. We do not also suggest that all tool processing for this period is limited to areas of parietal cortex and starts at 200 ms. The relation to “Meaningful category processing” model extracts only activity that is common for all tools and represents “extra” processing which leads to their delineation as a unified category of objects. In this respect, it is worth to note that if some intermediate analysis take place (results are not shown) where each individual image of tool is related to a degraded “Meaningful category processing” model (which does not require similarity within tool category) and then the results are averaged across tool exemplars, the resulting spatial map of active regions is more extensive including some ventral regions but preserves dorsal prevalence. In the next subsection, we discuss distinctions in different category binding mechanisms for faces and tools, while here we point out that in both cases categorization stage ends by about 240 ms after stimulus onset, when it is replaced by supra-category processing.

Here we use the term “supra-category” to name a stage operating in common for all category representation of meanings whether they be pictorial, symbolic, or verbal. A recent study has shown that after category selective encoding in posterior areas, activity was shifted to anterior temporal, parietal and frontal regions and was not specialized for category (Linden et al., 2012). One possible explanation for the dissociation may be that the sustained but not selective activation in widely distributed regions across parietal, temporal, and prefrontal cortex is related to multimodal concept processing (Visser et al., 2012). The ultimate outcome of this stage is to perform an organism’s response such as object naming (Grèzes and Decety, 2002), dependent action (Beets et al., 2010), operating within working memory (Linden et al., 2012), or introspection (Frässle et al., 2014). A recent study has shown (Beets et al., 2010) that the stability of a percept is affected by percept-related actions in which congruent movements stabilize the percept and incongruent movements destabilize the percept. This phenomenon gives explanation as to why we chose the naming paradigm instead of passive viewing for perception investigation, despite the active debates related to it applicability. We believe that any act of meaningful perception ends with an active response, either overt or covert, and so verbal response in our study was a small fee for explicit control of stimulus recognition.

### Distinguishing Characteristics of Feature Binding Mechanisms for Face and Tool Perception

We asked which differences in cortical topography are related to perceptual categorization and which of them reflect later activation of response-related brain processes. To answer these questions, we separated the spatiotemporal clusters of neuronal activity corresponding to different stages of visual object identification. We then applied a strict model (type 3 model, see **Figure 1**) to distinguish between faces and a group of combined tool and nonsense stimuli while requiring that there was similarity within each group (in particular, we required that processing of tools was not distinguishable from that of nonsense stimuli). We have detected corresponding spatiotemporal patterns in ventral occipito-temporal regions, the most salient in bilateral IO and FG areas, during an interval from 140 to 170 ms post-stimulus presentation. The time window of these patterns are within the interval expected to represent the perceptual categorization stage so we can ascribe them to a mechanism of face-specific feature binding.

When we applied similar type 3 model distinguishing between tools and a group of combined face and nonsense stimuli we have found tool-specific patterns in the regions, namely leIPS, leMTp (with adjacent STSp) and bilateral VPM, that taken together covers the well-known network of tool preferred areas (Beauchamp and Martin, 2007). However, only the pattern located to the leIPS occurred within the perceptual categorization stage at time window 210–220 ms and should be related to the tool-specific feature binding activity. By naming the third stage as “supra-categorical” we do not intend to state that some category dependent activity does not still occur after 250 ms. What we suggest here is that the activity occurring outside the second stage does not contribute into the categorical feature binding processes and so the functions of the late tool preferring regions, in particular MTp and VPM, should be associated with response-related activity such as active report or introspection (e.g., Frässle et al., 2014).

The analysis of spatiotemporal characteristics of the feature binding related activity patterns reveals that, firstly, the locations of processing for faces and tools have a pronounced bias to ventral and dorsal visual streams respectively; and secondly, the latency of a tool-specific processing is later than for face-specific processing. In what follows, we suggest that this dissociation in time and space does not mean an existence of specialized brain modules for particular category processing but reflects differential involvement of regions depending on the depth of experience with the objects of particular category.

For a long time, holistic processing was used to explain what makes face recognition special. In a recent study using transcranial magnetic stimulation technique it was shown that rIO region of ventral visual stream (right occipital face area in the original notation) is causally implicated in the type of holistic detection that is required for perception of Mooney figures and that such role is not face-selective (Bona et al., 2016). In contrast, rIO does not appear to play a causal role in detection of shapes based on bottom-up integration of local components, demonstrating that its involvement in processing of non-face stimuli is specific for holistic processing. In our study, we observed that IO regions were the most prominent areas revealing face specific processing while their activity did not differentiate between tools and nonsense stimuli. Altogether these results indicate that information in IO areas is mandatory for holistic processing for presumably any category of perceptual objects, but this with a particularly increased demand of this area is required to drive the emergence of a whole percept from face stimuli.

The straightforward consequence of these findings is that the activation in some ventral visual stream regions is rather holistic-specific than face-specific and could be caused not by an appearance of a face *per se* but some characteristics of face, which other objects also possess but to a lesser degree. In order to gain an insight regarding the origins of this property, it is useful to summarize findings from experimental studies using a composite task paradigm (Richler and Gauthier, 2014). The essence of the paradigm is that holistic processing is testing by indexing the extent to which participants can ignore irrelevant information within a whole face and selectively attend to face parts. First of all, it was shown that holistic processing is caused by an influence of processes that are automatically and mandatorily recruited for the aligned faces and lead to failures of selective attention (Richler et al., 2012). Second, a number of studies have shown (Gauthier et al., 2000; Bukach et al., 2010; McGugin et al., 2014) that expertise with non-face objects can lead to holistic processing, with gradually increasing activity of face-selective areas in the ventral visual stream. Altogether these results provide evidence that the property that governs the recruitment of ventral regions into processing, is determined by a depth of experience with the object and its measure represents information relevance, different from that identified by an online task control. We suggest that this measure corresponds to a module of a vector in neural value-dependent reference space (Edelman and Tononi, 2000).

We can attempt to summarize our findings in the following information integration scheme (describing the categorization stage only). Stimuli of both categories start to be processed by means of value-dependent mechanism in the areas of ventral stream. Different values for a criteria of information integration can act in parallel by independent operators. Each operator performs only one task, in that it detects elements within an incoming sensory flow that could be assimilated according to the corresponding value. A similar scheme in some different context has been introduced by Houtkamp et al. (2003) who have called it “figure-background segregation.” On each level of hierarchy most “fed” values are naturally selected (Edelman, 1993) to provide input to the higher levels. Hierarchical structure of operators ensures fast convergence to a structure of high-level values, which defines the meaning of the object category, in our case, faces. In the case of tools, the value-dependent mechanism cannot extract sufficient information from sensory flow for the unambiguous bias of competition up to the highest level of the hierarchy. Instead, sensorimotor-based mechanisms are recruited to solve the part-to-part problem of sensory binding by using intermediate representations allocated by value-dependent processing. In this case the emergence of meaning is achieved through the additional integration of sensory input based on sensorimotor associations that have been learnt through experience, leading to an increased time of binding processing when compared to categories of objects possessing high subjective value.

### Value-Dependent and Sensorimotor-Based Feature Binding Mechanisms Evoke Different Perceptual Learning Effects for Faces and Tools

Multiple repetitions of the same stimuli in the present study acted to reduce complexity of the bitonal images recognition task. The hypothesis of the existence of two distinct feature binding mechanisms predicts oppositely directed changes of corresponding specific activities for faces and tools. The perceptual learning of value-dependent mechanism results in a change of the dynamical system’s control parameters in order to tune them for better matching to stimulus influence. An extremely simple but common example of an oscillator gives a model for what is happening here. The learning (of fictional “educable” oscillator) changes its natural frequency in such a way that it coincides with the periodicity of the external force and oscillator gets the opportunity to better assimilate the energy of this force. In a similar way, increased synchronization and subsequent intensification of category-specific activity can be expected in the case of perceptual learning of value-dependent mechanism. On the contrary, sensorimotor mechanism guides execution of internally driven part-to-part integration task and a learning reduces the neural load of this task through optimization of synaptic pathways.

Therefore, the ventral value-dependent system activity should increase as the stimuli become easier to recognize. In contrast, dorsal sensorimotor-based system should adapt to the task by learning the solution, requiring less efforts and therefore a reduction in the activity. This is exactly what we observed in the study when we were investigating repetition dependent behavior upon the responses for face and tool specific spatiotemporal patterns. We saw that the amplitude of neuronal activity in rIO and rFG in the time window of 140–170 ms during face stimuli processing gradually increased, whilst the amplitude of neuronal activity in leIPS in time window 210–220 ms during tool stimuli processing decreased for the entirety of the first experimental section.

Repetition suppression is a ubiquitous effect in neuroimaging studies. It even gives a ground for a “adaptation paradigm” which is based on the notion that only those sources of signal that are critically involved in the processing of a stimulus will show suppression to repeated presentations of that stimulus (e.g., Avidan et al., 2002). Repetition amplitude enhancement is a rarer phenomenon. Firstly, we point out that the repetition conditions in our experiment were different from that which are common in repetition suppression paradigms. We did not present the same image (or images of the same category) in a row but mixed it in pseudorandom order with the remainder of the images, such that between two presentations of the same stimulus an average of nine other image presentations took place. In the similar conditions, Schendan and Kutas (2003) found reliable repetition enhancement effects at 148 ms, where the vertex P150 was more positive for repeated objects than for novel objects. Furthermore, Morel et al. (2009) using combined electro-encephalography and MEG recording have demonstrated that face-selective components at 170 ms after stimulus presentation showed repetition enhancement selective to neutral faces, with greater amplitude for emotional than neutral faces on the first but not the second presentation. Authors have suggested that these differential repetition effects may reflect valence acquisition for the neutral faces due to repetition. Such a combined influence of emotion- and learning-related factors of face encoding speaks in favor of the hypothesis about value-dependent nature of the experience that shapes category of faces.

Complementary evidence of the relation of an object’s recognizability with an increased activity of the value system during the perceptual categorization stage has been reported in the literature. Anderson and Phelps (2001) have applied a variant of the attentional blink paradigm and have shown that emotional targets were considerably more detectable than neutral ones, in an effect that required the intact amygdala. In electroencephalographic experiments it is difficult to measure the activity of the amygdala due to its small size and deficiency of laminar organization (see, however, Streit et al., 2003; Cornwell et al., 2008), therefore cortical regions supporting emotional value should be considered. Several lines of evidence from lesion, electrophysiological and anatomical studies indicate that the anterior insula in the right hemisphere aids in the evaluation of stimulus salience (Eckert et al., 2009). In the present study, we observed that activity in the right insula during the time window 140–170 ms significantly distinguished face category but did not distinguish the tool category from nonsense stimuli. A similar result showing face specific activity in bilateral insula ∼140 ms after stimulus onset was obtained in the recent MEG study of face and body perception (Meeren et al., 2013). Finally, a remarkable result indicating that naked bodies in contrast to clothed ones elicited N170 even larger than faces did (Hietanen and Nummenmaa, 2011) has demonstrated that an elevated emotional significance of stimuli can even change common face preferring behavior of the N170 component. Altogether, this results offer a support to the notion that values assigned to the incoming information boost integration processes in the ventral visual stream and increase the likelihood of the emergence of a meaningful and reportable percept.

### Summary

In this study, we made MEG recordings to which we applied a novel region-based multivariate pattern classification approach. Using this tool in combination with RSA we have extracted activity associated with three qualitatively distinct processing stages of visual perception. In contrast with the regions that have been reported elsewhere for differential activation recognition of faces and tools, we presented differences in processing in terms of both precise timing and brain regions involved. We found that these differences occur both at the categorical stage, as well as during a later stage of supra-categorical processing. Given the long lasting discussion on the attribution of the observed differences to a perception *per se*, or higher-tier cognitive processes (Jolicoeur et al., 1984; Chan et al., 2011; Frässle et al., 2014; Reichert et al., 2014), we believe that obtained findings are of importance. Moreover, we provided evidence for the opposing action of stimulus repetition effect for faces and tools, i.e., repetition suppression for tools and repetition enhancement for faces. Since both effects were presented at the categorization stage and were expressed in the critical nodes of the respective categorization networks—FG and IO for faces, and IPS for tools—they further support the claim that faces and tools are processed differently already at the “intermediate” categorization stage. We have discussed these results in a framework that assumes the existence of two different binding mechanisms for categories that are shaped predominantly by either value-dependent or sensorimotor experience.

## Author Contributions

VK contributed to study conception and design, acquisition of data, analysis methods development, analysis and interpretation of data, drafting of manuscript, critical revision. AN contributed to acquisition of data, drafting of manuscript, critical revision. TS contributed to study conception and design, drafting of manuscript, critical revision.

## Conflict of Interest Statement

The authors declare that the research was conducted in the absence of any commercial or financial relationships that could be construed as a potential conflict of interest.
